# Long‐term exposure of sucralose induces neuroinflammation and ferroptosis in human microglia cells via SIRT1/NLRP3/IL‐1β/GPx4 signaling pathways

**DOI:** 10.1002/fsn3.4488

**Published:** 2024-09-23

**Authors:** Ceyhan Hacioglu

**Affiliations:** ^1^ Department of Biochemistry, Faculty of Pharmacy Düzce University Düzce Turkey; ^2^ Department of Medical Biochemistry, Faculty of Medicine Düzce University Düzce Turkey

**Keywords:** artificial sweeteners, ferroptosis, microglia, neuroinflammation, NLRP3, sirtuins, sucralose

## Abstract

Microglia serve as the primary defense mechanism in the brain. Artificial sweeteners are widely used as dietary supplements, though their long‐term effects remain uncertain. In this study, we investigated the effects of sucralose on microglia during prolonged exposure via the neuroinflammatory and ferroptosis pathways. Initially, human microglial clone 3 (HMC3) cells were exposed to sucralose (0–50 mM) for 24, 48, and 72 h to investigate the short‐term effects. Subsequently, HMC3 cells were treated with 1 mM sucralose for 7, 14, and 21 days to examine long‐term effects. We measured levels of interleukin‐1β (IL‐1β), NOD‐like receptor protein 3 (NLRP3), 8‐hydroxydeoxyguanosine (8‐OHdG), Sirtuin‐1 (SIRT1), glutathione peroxidase‐4 (GPx4), reduced glutathione (GSH), malondialdehyde (MDA), ferrous iron (Fe^2+^), and caspase 3/7. Additionally, we analyzed the impact of sucralose on cell morphology, migration, and expression levels of IL‐1β, NLRP3, SIRT1, and GPx4. Sucralose inhibited cell viability and proliferation in HMC3 cells in a concentration‐ and time‐dependent manner and induced membrane and nuclear abnormalities. Moreover, sucralose significantly reduced the cell migration rate. Long‐term sucralose treatment decreased Fe^2+^, GPx4, GSH, and SIRT1 levels in HMC3 cells while increasing IL‐1β, MDA, NLRP3, 8‐OHdG, and caspase 3/7 activity. Sucralose treatment also enhanced microglial activation and neuroinflammation by upregulating IL‐1β and NLRP3 and downregulating SIRT1 and GPx4, thereby inducing ferroptosis and suppressing cell viability. Consequently, high concentrations or long‐term sucralose treatment may induce neuroinflammation and ferroptosis by targeting the SIRT1/NLRP3/IL‐1β/GPx4 pathway in HMC3 cells.

## Graphical abstract

Long‐term sucralose treatment may induce neuroinflammation and ferroptosis. Sucralose may trigger SIRT1/NLRP3/IL‐1β/GPx4 pathways. Artificial sweeteners may affect brain health

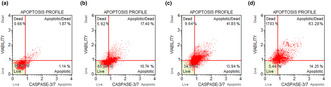

## 1 INTRODUCTION

Artificial sweeteners are commonly utilized as sugar substitutes to sweeten beverages and foods, particularly by individuals seeking to reduce their calorie intake, including those with diabetes or those aiming for weight management (Liu et al., 2021). These high‐intensity sweeteners provide a sweet taste similar to sucrose but do not undergo metabolism in the body, resulting in no calorie intake. One such widely approved non‐caloric sweetener is sucralose (chemical name: 1,6‐dichloro‐1,6‐dideoxy‐ß‐d‐fructofuranosyl‐4‐chloro‐4‐deoxy‐α‐d‐galactopyranoside). Sucralose is derived from sucrose through the selective replacement of three hydroxyl groups with chlorine atoms. It possesses a sweetness potency approximately 600 times that of sucrose, allowing for the use of very small amounts to sweeten foods and beverages. Importantly, unlike sucrose, sucralose is not digested or metabolized for energy in the body. As a result, it provides no calories when consumed and does not impact blood glucose levels. Due to its non‐metabolizable nature, sucralose has become widely used in a variety of foods and beverages as a safe alternative to sugar (AlDeeb et al., 2013). Initially, these sweeteners were considered metabolically inert, meaning they were believed to have no impact on the body (Magnuson et al., 2017). However, further research revealed that the gustatory mechanism, responsible for the sense of taste, is not limited to the tongue alone but is also present in the small intestine (Schiffman & Rother, 2013). Studies on animals have shown that large quantities of sucralose within the gastrointestinal tract can lead to gastrointestinal disturbances and histopathological changes in the colon of rats (Abou‐Donia et al., 2008). These effects may include alterations in the gut microbiota, changes in gut barrier function, and inflammation in the colon. Recently, Bagga et al. (2017) reported that sucralose can potentially serve as a contrast agent for clinical magnetic resonance imaging (MRI) applications due to its low molecular weight, lipophilic character, ability to pass through the blood–brain barrier, and accumulation in tumor tissue without being metabolized. These results have raised concerns and suspicions about the potential metabolic effects of sucralose.

Ferroptosis is a type of cell death that depends on iron and is characterized by the accumulation of lipid peroxidation products (Chen et al., 2020). Iron is an essential trace element in the body, playing crucial roles in various cellular processes, including oxygen transport, DNA biosynthesis, and ATP generation (Winter et al., 2014). The regulation of iron metabolism and the maintenance of cellular redox balance are critical in preventing ferroptosis. Proper regulation of cellular iron is crucial for maintaining brain function (Rauf et al., 2023). The iron regulatory protein 1 (IRP1) plays a central role in regulating the expression and activity of both divalent metal transporter 1 (DMT1) and ferroportin (Salvador, 2010). Inflammatory cytokines such as tumor necrosis factor‐α (TNF‐α) and interleukin‐6 (IL‐6) can influence iron handling in microglia, leading to iron sequestration within the cells (Rathore et al., 2012). The process of iron sequestration is associated with the upregulation of DMT1 in microglia. The presence of IL‐6 and TNF‐α can increase the expression of hepcidin in microglia, which further affects iron levels within the cells. This leads to an increase in iron influx and a decrease in iron efflux, ultimately resulting in iron accumulation within microglia. Inflammatory processes can modulate the expression of key iron‐regulatory proteins, influencing iron uptake and retention within microglia. These findings highlight the complex relationship between neuroinflammation and ferroptosis in microglia that is not yet fully understood.

Sirtuins (SIRTs) play crucial roles in various cellular processes, including gene silencing, stress response, apoptosis, senescence, aging, and inflammation (Houtkooper et al., 2012). SIRT1, a member of the Sirtuin family, has been extensively studied for its neuroprotective effects and antioxidant properties. SIRT1 has demonstrated significant antioxidant activity and provides neuroprotection (Yao & Rahman, 2012). One mechanism by which SIRT1 exerts its antioxidant effects is by downregulating the expression of certain proteins involved in oxidative stress. Additionally, studies have reported that SIRT1 activation can also protect against ferroptosis, a form of cell death characterized by iron‐dependent lipid peroxidation (Yuan et al., 2022). SIRT1 activation increases levels of glutathione peroxidase 4 (GPx4) and ferroptosis suppressor protein 1 (FSP1) after brain injury, thus providing protection against ferroptotic cell death. SIRT1 inhibits lipid peroxidation, a key characteristic of ferroptosis. Furthermore, SIRT1 reduces the expression of acyl‐CoA synthetase long‐chain family member 4 (ACSL4) and level of malondialdehyde (MDA) (Chen et al., 2022). By decreasing ACSL4 expression and MDA levels, SIRT1 inhibits the production of lipid peroxides, thereby protecting cells from ferroptotic damage.

The traditional view of the central nervous system (CNS) as an immunologically privileged region, where inflammation was considered a passive response to neuronal damage, has evolved in recent years. Within the CNS, microglia are the principal innate immune cells, constantly surveying the nearby environment through pattern‐recognition receptors such as Toll‐like receptors and NOD‐like receptors (NLRs) (Walsh et al., 2014). Recently, a novel inflammatory pathway called inflammasomes has been identified in neurodegeneration. Research suggests that NOD‐like receptor protein 3 (NLRP3) inflammasomes may play a crucial role in mediating inflammatory responses and inducing cellular damage in this condition (de Zoete et al., 2014). NLRP3 inflammasomes are a well‐characterized subset of the NOD‐like receptor family, comprising NLRP3, an apoptosis‐associated speck‐like protein containing a caspase activation recruitment domain (ASC), and precursor caspase‐1 (pro‐caspase‐1). They are implicated in neuronal damage by triggering the release of proinflammatory cytokines such as IL‐1β and IL‐18 through the activation of caspase‐1. Subsequently, these cytokines contribute to the initiation and amplification of the inflammatory responses in the affected area (Blevins et al., 2022). Notably, the NLRP3 inflammasome is not restricted to immune cells but has also been detected in various other cell types responsible for diverse physiological functions (Tőzsér & Benkő, 2016). These include muscle cells, neurons, and endocrine cells, indicating that the inflammasome may have a broader role beyond the traditional immune response. Moreover, it has been emphasized that autophagy, oxidative stress, and chronic inflammation mechanisms lead to microglial activation and are focal points in the progression of neurodegenerative disorders (Kabir et al., 2022; Uddin et al., 2018).

In this study, we examined the effects of short‐term sucralose treatment on the human microglial clone 3 (HMC3) cell line, as well as the effects of long‐term sucralose exposure on neuroinflammation and ferroptosis pathways. We analyzed the subchronic effects of sucralose in HMC3 cells by measuring levels of Fe^2+^, IL‐1β, NLRP3, 8‐hydroxydeoxyguanosine (8‐OHdG), SIRT1, GPx4, reduced glutathione (GSH), and MDA. Additionally, we investigated the effects of sucralose on the expression levels of IL‐1β, NLRP3, SIRT1, and GPx4. Furthermore, we examined the effects of sucralose treatment on nuclear structure and cell morphology in HMC3 cells.

## 2 MATERIALS AND METHODS

### 2.1 Cell culture and sucralose treatment

The HMC3 cell line used in this study was obtained from the American Type Culture Collection (ATCC). HMC3 cells were originally generated by introducing the large T antigen of the simian virus 40 into human embryonic brain‐derived macrophages. The cells were cultured in Dulbecco's Modified Eagle's Medium (DMEM; Thermo Fisher Scientific) supplemented with 10% fetal calf serum (Sigma‐Aldrich) and 100 units/mL (U/mL) penicillin/streptomycin (Invitrogen). The cells were incubated in a humidified atmosphere with 5% CO_2_ at 37°C.

Once the HMC3 cells reached 80% confluence, they were detached from the culture plate using 0.25% Trypsin–EDTA (Sigma‐Aldrich). Following detachment, the cells were seeded onto new culture plates for maintenance or specific experiments. During regular maintenance, the cells received fresh medium every 3–4 days to ensure an adequate supply of nutrients and to maintain optimal growth conditions. For experimental treatments, the cells were plated in 24‐well plates at a density of 2 × 10^5^ cells per well and allowed to grow for at least 24 h before being treated with sucralose for further analyses.

To prepare the 0.25 M sucralose (69293; Sigma‐Aldrich) stock solution, DMEM was used as the solvent. After preparing the stock solution, it was filtered through a 0.22 μm pore membrane to remove any particulate matter or contaminants. The stock solution was then further diluted with complete medium to achieve the final desired concentrations (0, 1, 5, 10, 20, and 50 mM) for the experiments. The pH of the diluted sucralose solution was adjusted to 7.4 to ensure it was suitable for the experimental conditions.

### 2.2 Cell viability and proliferation

To determine cytotoxic sucralose concentrations (short‐term treatment), the cells were treated with the experimental substances at different time points (24, 48, and 72 h). The study conducted by van Eyk (2015) found that in vitro exposure to concentrations of artificial sweeteners greater than 10 mM resulted in alterations in cell morphology, attributed to high osmolarity effects. Therefore, to determine the long‐term effects of sucralose, we chose a 1 mM sucralose concentration for 21 days. After the treatment period, 100 μL of MTT (3‐(4,5‐dimethylthiazol‐2‐yl)‐2,5‐diphenyltetrazolium bromide) solution was added to each well of the culture plate. The cells were then incubated at 37°C for 4 h. During this incubation, viable cells convert the yellow MTT dye into purple formazan crystals through mitochondrial enzyme activity. After the 4‐h incubation, 100 μL of dimethyl sulfoxide (DMSO) was added to each well to dissolve the formazan crystals formed by viable cells. The absorbance of the dissolved formazan was measured at 570 nm using a microplate reader (BioTek). The measurements were taken for each treated sample, and a blank (medium without cells) was included to account for any background absorbance. Cell viability was calculated using the following formula:

$$\text{Cell viability}\;\left(\%\right)=\left[\left(\text{Absorbance of the treated cells}\right)-\left(\text{Absorbance of the blank}\right)\right]/\left[\left(\text{Absorbance of the control cells}\right)-\left(\text{Absorbance of the blank}\right)\right]\times 100$$

The 5‐bromo‐2′‐deoxyuridine (BrdU) incorporation assay is based on the incorporation of a thymidine analog into the DNA of actively dividing cells during the S phase of the cell cycle. In this study, cells were treated with various concentrations of sucralose for different time periods: 24, 48, 72 h, 7, 14, and 21 days. During long‐term exposure, the cell culture medium was replaced with fresh medium containing sucralose in 72‐h cycles for 21 days. To determine cell proliferation, the BrdU cell proliferation assay kit (2750; Sigma‐Aldrich) was used. Following the manufacturer's instructions, cells were labeled with BrdU, and after an appropriate incubation period, the cells were fixed and processed to allow for the detection of BrdU incorporation. The absorbance values of untreated and treated cells were then measured at 450 nm using a microplate reader.

### 2.3 Imaging cellular morphology

HMC3 cells were seeded at a density of 3 × 10^3^ cells per well and treated with 1 mM sucralose for 7, 14, and 21 days. After the treatment period, the cells were washed with phosphate‐buffered saline (PBS) to remove any residual sucralose. Subsequently, the HMC3 cells were examined under an inverted microscope to visualize the cell structure.

### 2.4 Cell migration analysis

To assess the impact of sucralose (1 mM) on cell migration and wound healing, a confluent monolayer of HMC3 cells was grown in a 6‐well plate and subjected to a scratch assay. The scratch was made by carefully dragging a 200‐μL pipette tip across the cell monolayer, creating a well‐defined wound area. The cells were then washed twice with PBS to eliminate any debris. To minimize the influence of cell proliferation on the wound healing process, the cells were cultured in a medium containing only 1% FBS, which restricts cell growth. Following the creation of the wound, the cells were treated with sucralose. Images of the wound area were captured at 0 h (immediately after scratching) and at 48 and 72 h after treatment. This was done using an Oxion Inverso microscope equipped with a CMEX‐5 Pro camera at 20× magnification. The size of the wound was quantified using ImageJ software.

### 2.5 Biochemical analyzes

For the biochemical analyses, HMC3 cells were grown in a 96‐well plate at a density of 5 × 10^3^ cells per well. Once the cells reached sufficient confluence, the plate was divided into four groups: control (I), group treated for 7 days (II), group treated for 14 days (III), and group treated for 21 days (IV). Each group was treated with 1 mM sucralose. After the treatment period, the cells were washed with cold PBS at pH 7.0 to remove any residual medium. The cells were detached from the wells using trypsin and collected by centrifugation at 1000 ***g*** for 5 min at 4°C. After centrifugation, the cells were washed three times with cold PBS to remove any remaining traces of medium and trypsin. The resuspended cells were then incubated in radioimmunoprecipitation assay (RIPA) lysis buffer (Santa Cruz) for half an hour with intermittent shaking at 4°C. Following cell lysis, the lysate was subjected to centrifugation at 16,000 ***g*** for 10 min at 4°C. The proteins present in the supernatant were collected for further analysis. Protein levels in cell lysates were determined to be 1 mg/mL using the BCA Assay Kit (E‐BC‐K318‐M; Elabscience), and this amount was used for each sample during enzyme‐linked immunosorbent assay (ELISA) measurements.

To quantify the levels of IL‐1β, NLRP3, 8‐OHdG, SIRT1, MDA, GSH, GPx4, and Fe^2+^ in the cell lysates, commercially available ELISA kits (RAB0273, EH4202, ab201734, RAB1699, MAK085‐1KT, MAK364, MBS2000338, and E‐BC‐K881‐M, respectively) were used. Each ELISA kit consists of a 96‐well plate pre‐coated with immobilized antibodies specific to the target proteins. The cell lysate samples obtained from the previous steps were added to the wells of the ELISA plate. The target proteins in the cell lysates bind to the immobilized antibodies on the surface of the wells, forming a protein‐antibody complex. After an incubation period, the plate was washed to remove any unbound substances. Specific detection antibodies were then added to each well. The plate was incubated again to allow the detection antibodies to bind to the protein‐antibody complex. Following this incubation, another washing step was performed to remove any unbound detection antibodies. A substrate solution was added to each well, which reacted with the enzyme linked to the detection antibodies, resulting in a color change. The color change produced in each well is proportional to the amount of the target protein present in the cell lysate. The color intensity was measured using a microplate reader.

### 2.6 Quantitative real‐time polymerase chain reaction analysis

To assess the expression levels of IL‐1β, NLRP3, SIRT1, and GPx4, total RNA was isolated from both the treated and untreated cell groups using TRIzol® Reagent, following the manufacturer's protocol. Subsequently, the extracted RNA was subjected to reverse transcription using the SuperScript™ IV One‐Step RT‐PCR System. In this step, 1 μg of RNA from each group was utilized. For the amplification of complementary DNAs (cDNAs), the StepOnePlus™ Real‐Time PCR System was employed, along with SYBR Green Master Mix. The primer sequences used in the amplification reactions for IL‐1β, NLRP3, SIRT1, and GPx4 were as follows: IL‐1β forward primer 5′‐ACG AAG GCC TAA GAC AGA GCC GT‐3′, IL‐1β reverse primer 5′‐GCA GTA CAT AGG AAG CCG GGA‐3′; NLRP3 forward primer 5′‐GCA GCA CGT TAA TGC CGA GA‐3′, NLRP3 reverse primer 5′‐GCC TCA AGG ACG ACT CGT AGG‐3′; SIRT1 forward primer 5′‐TAG CCT TGT CAG ATA AGG AAG GA‐3′, SIRT1 reverse primer 5′‐ACA GCT TCA CAG TAC ACT TTG T‐3′; GPx4 forward primer 5′‐AGA GAT CAA AGA GTT CGC CGC‐3′, GPx4 reverse primer 5′‐TCT TCA TCC ACT TCC ACA GCG‐3′. The PCR amplification was carried out under the following cycling conditions: 50°C for 2 min, 95°C for 10 min, followed by 40 cycles of 95°C for 15 s and 60°C for 1 min. To normalize the data, the expression levels were compared to the housekeeping gene β‐actin. The relative expression of mRNA was determined using the $2^{-∆∆C_{t}}$ method, which involves calculating the fold change in gene expression between the treated and untreated samples after normalizing to β‐actin.

### 2.7 Caspase‐3/7 activation analysis

The apoptotic status of cells treated with sucralose and the control group was analyzed using the Muse® Caspase‐3/7 kit (MCH100108). Caspase‐3/7 are key enzymes involved in the execution phase of apoptosis. To determine the different apoptotic cell populations, the dead cell marker 7‐aminoactinomycin D (7‐AAD) was used, which measures cellular plasma membrane permeability. In the experimental procedure, a total of 5 × 10^3^ cells were treated with sucralose for 7, 14, and 21 days. After the incubation period, the cells were trypsinized and washed with PBS. Then, 5 μL of the Muse® Caspase‐3/7 working solution was added to 50 μL of the cell suspension. The mixture was incubated at 37°C for 30 min, allowing the Caspase‐3/7 probes to bind to active caspases in apoptotic cells. Next, 150 μL of the 7‐AAD dye was added to the cell suspension, and the solution was thoroughly mixed. 7‐AAD is a fluorescent dye that can enter cells with compromised plasma membranes, which is characteristic of dead or apoptotic cells. Finally, the cell suspension with both the Caspase‐3/7 probes and 7‐AAD dye was analyzed using the Muse® Cell Analyzer.

### 2.8 Statistical analysis

The experiments were conducted with a minimum of three replicates. The data obtained from the experiments are presented as mean values, with variability indicated by the mean ± standard deviation (SD). To compare the means of multiple groups, a two‐way analysis of variance (ANOVA) was used. Statistical analyses were performed using GraphPad Prism 8 software. A *p*‐value of less than .05 was considered statistically significant.

## 3 RESULTS

### 3.1 Short‐term effect on cell viability and proliferation of HMC3 cells treated with sucralose

**FIGURE 1:**
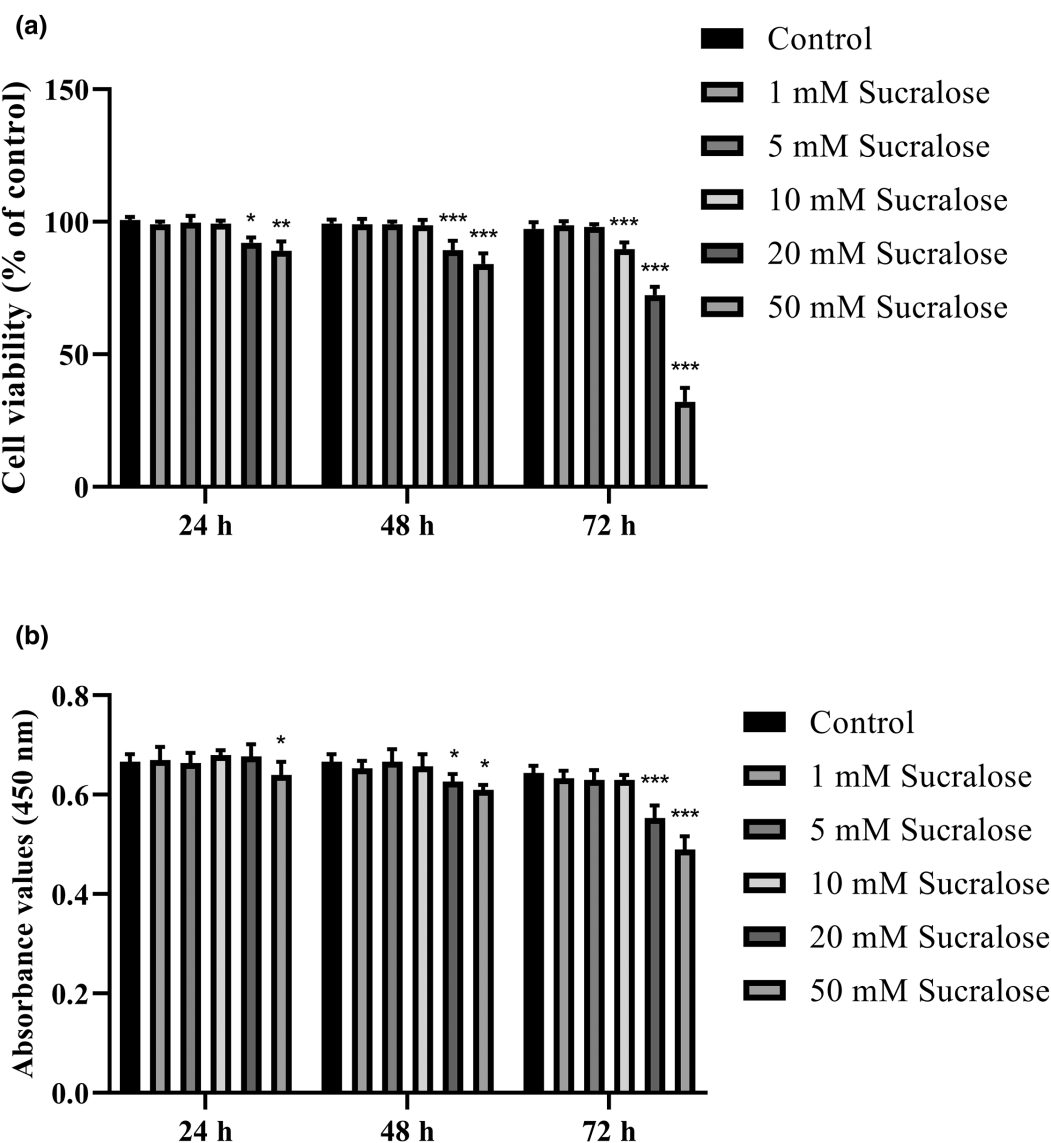
Sucralose treatment (in the range of 0–50 mM) affected cellular viability and proliferation in HMC3 cells up to 72 h. (a) MTT results in HMC3 cells; (b) BrdU incorporation in HMC3 cells. *p < .05, **p < .001, and ***p < .0001 vs. control group.

Figure 1 illustrates the effect of different concentrations of sucralose on the cell viability of HMC3 cells over various time periods (24, 48, and 72 h, short‐term treatment). The results demonstrate that sucralose treatment led to a decrease in cell viability in a concentration‐ and time‐dependent manner, albeit to varying degrees. When HMC3 cells were treated with low concentrations of sucralose (ranging from 1 to 10 mM), there was slightly higher cell viability compared to untreated cells, but this difference was not statistically significant (*p* > .05). In other words, low concentrations of sucralose did not significantly affect cell viability during the initial 24 h of treatment. However, as the concentration of sucralose increased above 20 mM, there was a notable decrease in cell viability by 7.3% (*p* = .0247 vs. control), and this decrease followed a dose‐dependent pattern. Furthermore, after 24 h of incubation, 50 mM sucralose treatment resulted in a significant reduction in HMC3 cell viability (13.5% of control, *p* = .0064).

As the incubation periods increased to 48 and 72 h, statistically significant differences in cell viability were observed in sucralose‐treated cells compared to the control group. This indicates that the detrimental effect on cell viability became more pronounced with longer exposure to sucralose. At 48 h, treatment with sucralose concentrations ranging from 1 to 10 mM did not significantly affect HMC3 cell viability compared to the control group (Figure 1a). However, exposure to a 20 mM sucralose concentration for 48 h led to a notable decrease in cell viability by 18.2% (*p* < .0001 vs. control). Furthermore, treatment with a 50 mM sucralose concentration for 48 h resulted in a significant reduction in HMC3 cell viability by 26.9% (*p* < .0001 vs. control). After 72 h of treatment, the impact of sucralose on HMC3 cell viability was even more prominent (Figure 1a). Sucralose concentrations ranging from 1 to 5 mM resulted in a slight reduction in cell viability, which was not statistically significant compared to the control group (*p* > .05). However, exposure to 10 and 20 mM sucralose concentrations for 72 h led to a significant decrease in cell viability by 19.2% and 30.5%, respectively (*p* < .0001 vs. control). Moreover, treatment with a 50 mM sucralose concentration for 72 h caused a substantial reduction in HMC3 cell viability by 64.6% (*p* < .0001 vs. control). Among all the tested concentrations, the most significant reduction in cell viability was observed after 72 h of treatment with a 50 mM sucralose concentration.

The results from the BrdU assay confirmed the antiproliferative effects of sucralose on HMC3 cells, further supporting the findings from the cell viability analyses. After 24 h of treatment, a 50 mM sucralose concentration significantly inhibited cell proliferation in HMC3 cells (8.6% of control; *p* = .0326), while lower concentrations (1, 5, 10, and 20 mM) did not show a statistically significant effect on cell proliferation (*p* > .05 vs. control; Figure 1b). At 48 h of treatment, the antiproliferative effects of sucralose became more evident. HMC3 cell proliferation was inhibited by 9.1% and 13.5% in response to 20 and 50 mM sucralose concentrations, respectively, compared to the control group (*p* < .05; Figure 1b). Similarly, after 72 h of treatment, sucralose exerted a concentration‐dependent antiproliferative effect on HMC3 cells. Treatment with 20 and 50 mM sucralose concentrations led to a significant suppression of cell proliferation by 18.6% and 35.2%, respectively, compared to the control group (*p* < .0001). These findings indicate that sucralose treatment has a negative impact on HMC3 cell proliferation in a concentration‐ and time‐dependent manner. Higher sucralose concentrations (20 and 50 mM) and longer exposure durations (48 and 72 h) resulted in more pronounced inhibition of cell proliferation, while lower concentrations (1, 5, and 10 mM) showed limited effects. The results suggest that prolonged exposure to high concentrations of sucralose may have detrimental effects on the proliferative capacity of HMC3 cells.

### 3.2 Long‐term effect on cell viability and proliferation of HMC3 cells treated with sucralose

**FIGURE 2:**
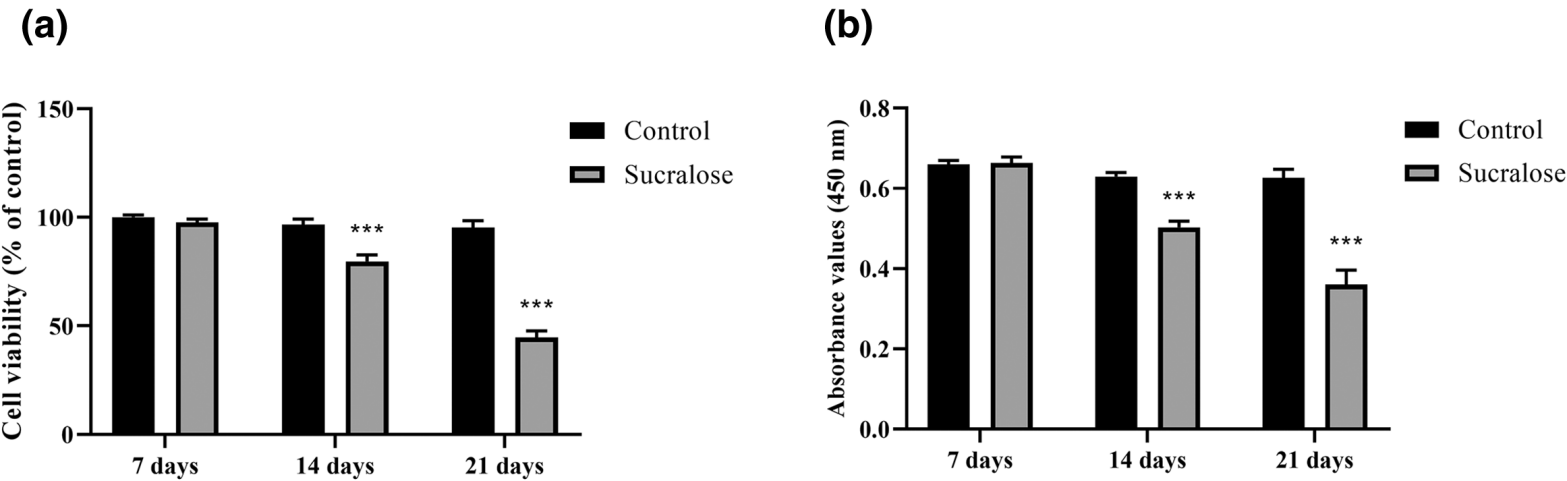
Long‐term sucralose treatment (1 mM) for 7, 14, and 21 days suppressed viability and proliferation in HMC3 cells. (a) MTT results in HMC3 cells; (b) BrdU incorporation in HMC3 cells. ***p < .0001 vs. control group.

According to the MTT assay results, Figure 2 shows the cell viability and proliferation outcomes after 7, 14, and 21 days of continuous exposure of HMC3 cell cultures to 1 mM sucralose starting from the moment of seeding. The findings indicate that the effects on cell viability and proliferation depend on the duration of the treatment. After 7 days of exposure to 1 mM sucralose, no significant differences were observed in the percentages of viable cells compared to the control group (*p* > .05 vs. control; Figure 2a). Cell viability remained comparable to that of untreated cells at this early time point. However, after 14 days of treatment, noticeable differences in cell viability were observed between the treated and untreated cells (Figure 2a). Cells exposed to 1 mM sucralose for 14 days showed a 21.4% decrease in cell viability compared to the control group (*p* < .0001), indicating potential viability‐suppressing effects of sucralose on HMC3 cells after prolonged exposure. Furthermore, after 21 days of continuous treatment with sucralose, cell viability was even lower in the treated cells (54.7% of control, *p* < .0001; Figure 2a). The results suggest that longer exposure to sucralose for 21 days leads to a larger proportion of cells experiencing viability‐suppressing effects, resulting in reduced cell viability.

Similarly, according to the BrdU assay, exposure to 1 mM sucralose for 7 days did not cause a statistically significant decrease in HMC3 cell proliferation, whereas exposure to sucralose for 14 and 21 days resulted in a decrease in cell proliferation by 18.2% and 46.1%, respectively (*p* < .0001 vs. control; Figure 2b). Taken together, the results from Figure 2 suggest that the effects of sucralose on HMC3 cell viability and proliferation are time‐dependent. While no significant differences were observed after 7 days of exposure, prolonged treatment for 14 and 21 days resulted in decreased cell viability and proliferation, indicating potential antiproliferative effects of sucralose in HMC3 cells over time. These findings highlight the importance of considering the duration of sucralose exposure in understanding its impact on cell viability and proliferation in this cell line.

### 3.3 Effect of sucralose on cell morphology

**FIGURE 3:**
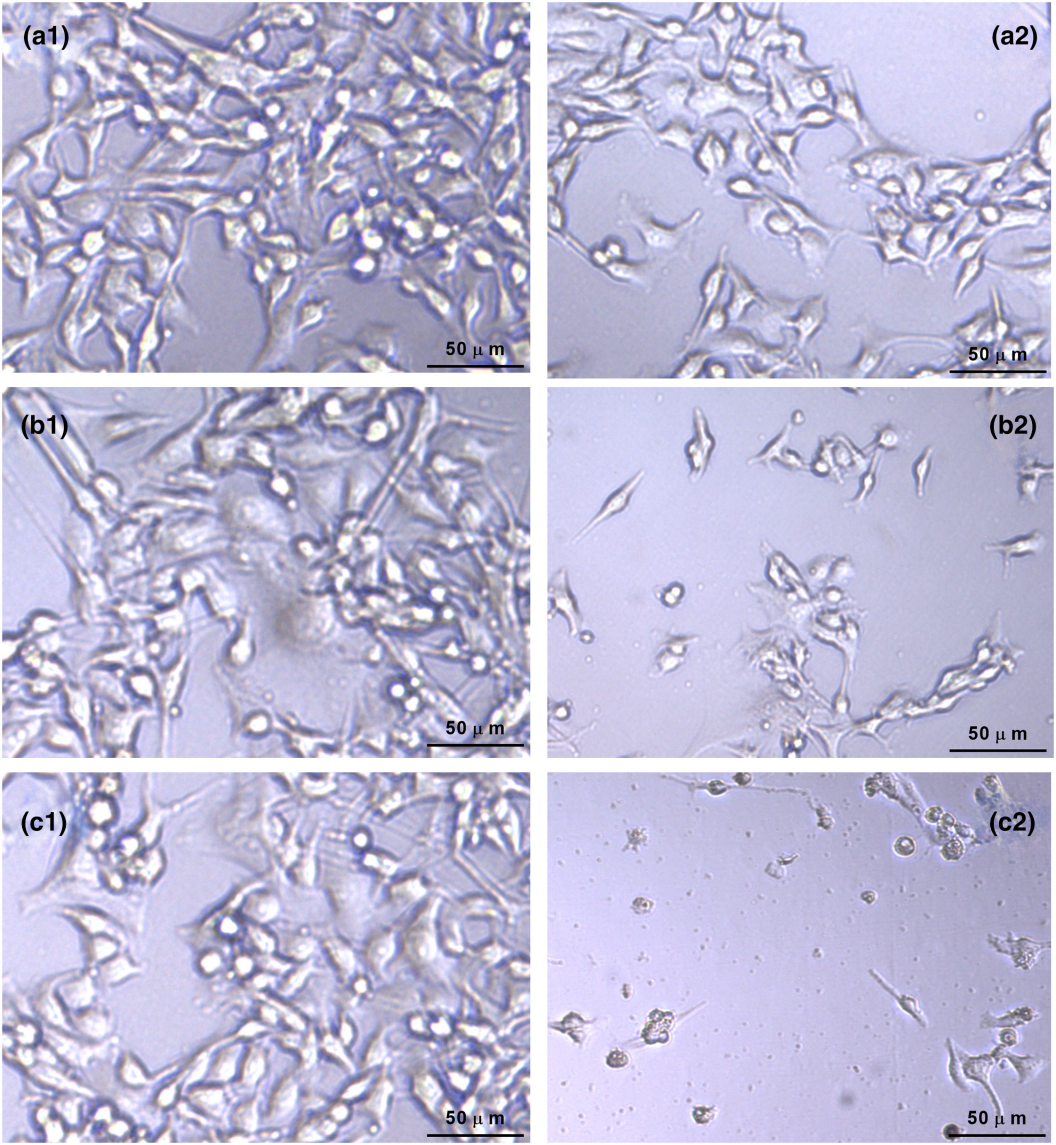
Effects of sucralose treatment on cell morphology in HMC cells. (a1–c1) Untreated HMC3 cells; (a2) HMC3 cells treated with 1 mM sucralose for 7 days; (b2) HMC3 cells treated with 1 mM sucralose for 14 days; (c2) HMC3 cells treated with 1 mM sucralose for 21 days.

By analyzing the inverted microscopy images, we assessed the cellular morphological abnormalities of HMC3 cells after exposure to sucralose for different durations (Figure 3). After 7 days of exposure to sucralose, no significant abnormalities were observed in the cells compared to the control group (Figure 3a,b). The cells exhibited a standard size and normal cytoplasmic morphology. However, as the treatment duration increased to 14 and 21 days, noticeable morphological degenerations were observed in the sucralose‐treated HMC3 cells (Figure 3c,d). These degenerative changes included round cell shapes and shrinking cells with reduced cytoplasmic volume. Moreover, the morphological differences in the HMC3 cells treated with sucralose were more prominent at 21 days compared to the 14‐day treatment period. This suggests that longer exposure to sucralose had a greater impact on cell morphology, possibly indicating a progressive cellular response to the treatment over time.

### 3.4 Effect of sucralose on cell migration

**FIGURE 4:**
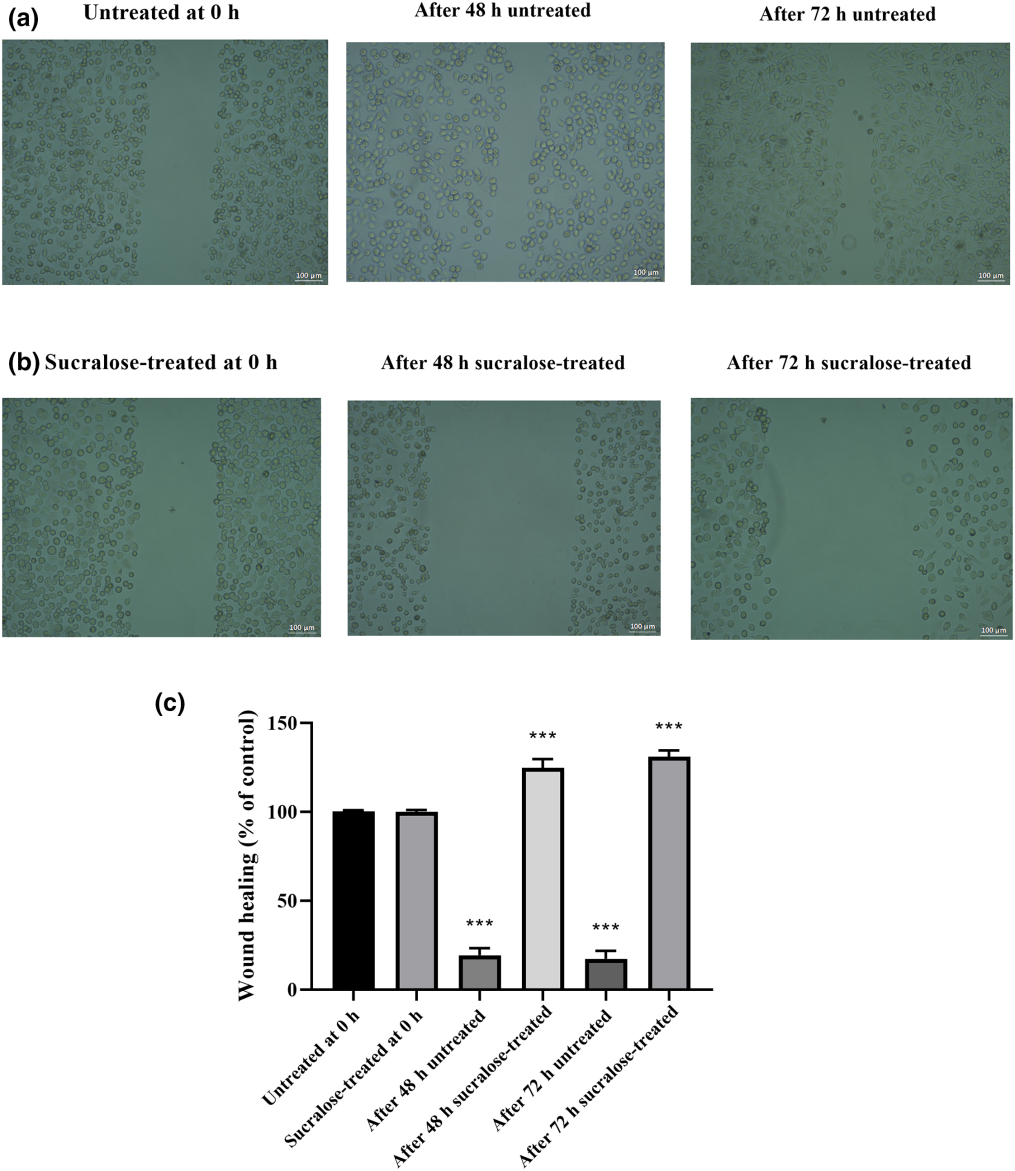
The impact of sucralose treatment on the cell migration rate in HMC3 cells. (a) Microscopic images of untreated cell migration in HMC3 cells. (b) Microscopic images of treated cell migration in HMC3 cells. (c) Cell migration rates in HMC3 cells. ***p < .0001 when compared to untreated/treated groups.

The results obtained from the wound‐healing assay (Figure 4a–c) clearly demonstrate that treatment with 1 mM sucralose significantly impacts the two‐dimensional migration ability of HMC3 cells. In the absence of sucralose treatment, a substantial portion of the wound area closed within 48 and 72 h, achieving closure rates of 75.91% and 83.62%, respectively (*p* < .0001, compared to untreated cells). Conversely, when subjected to 1 mM sucralose treatment, HMC3 cells exhibited a markedly reduced wound healing rate. After 48 h, the wound area of HMC3 cells increased by 12.57% (*p* < .001 vs. treated cells), while the wound area at 72 h increased by 18.84% (*p* < .001 vs. treated cells). These results strongly suggest that sucralose treatment hinders the migratory capacity of HMC3 cells, as evidenced by a reduction in the closure of the wound area.

### 3.5 Effect of sucralose on IL‐1β, NLRP3, 8‐OHdG, SIRT1, MDA, GSH, GPx4, and Fe^2+^ levels in HMC3 cells

**FIGURE 5:**
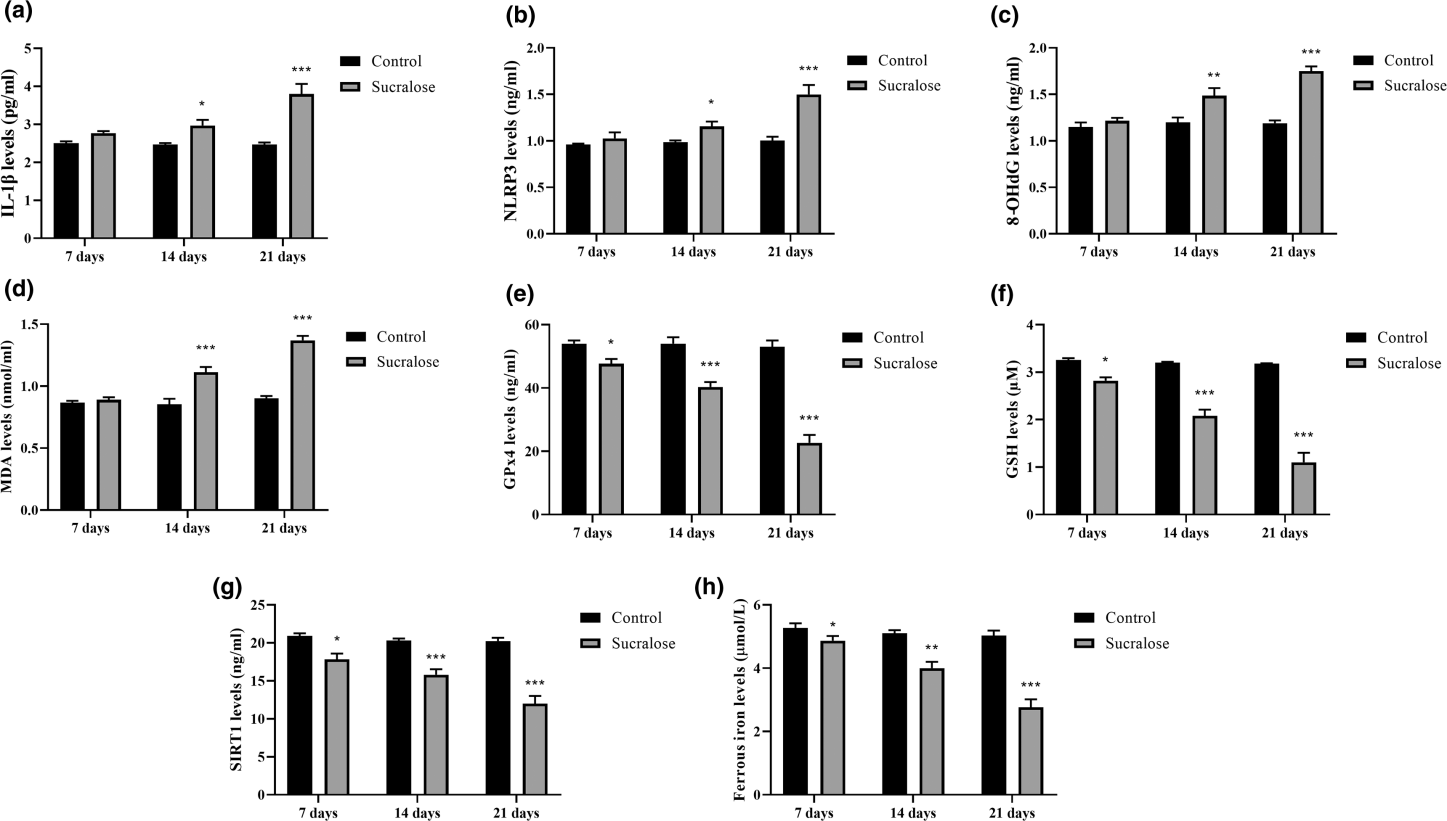
Long‐term sucralose treatment regulated IL‐1β, NLRP3, 8‐OHdG, SIRT1, MDA, GSH, GPx4 and Fe^2+^ levels in HMC3 cells. (a) IL‐1β levels in HMC3 cells; (b) NLRP3 levels in HMC3 cells; (c) 8‐OHdG levels in HMC3 cells; (d) MDA levels in HMC3 cells; (e) GPx4 levels in HMC3 cells; (f) GSH levels in HMC3 cells; (g) SIRT1 levels in HMC3 cells; (h) Fe^2+^ levels in HMC3. *p < .05, **p < .001, and ***p < .0001 vs. control group.

The findings presented in Figure 5 demonstrate that the treatment of HMC3 cells with sucralose had a notable impact on various molecular markers, including IL‐1β, NLRP3, 8‐OHdG, and SIRT1, along with factors associated with ferroptosis such as MDA, GSH, and GPx4. In HMC3 cells, the levels of IL‐1β, NLRP3, 8‐OHdG, and MDA exhibited a concentration‐dependent increase with sucralose treatment. Specifically, in HMC3 cells, the levels of IL‐1β increased by 7.3%, 15.1%, and 53.6% when treated with 1 mM sucralose concentration for 7, 14, and 21 days, respectively, compared to the control group (*p* = .1643, *p* = .0168, and *p* < .0001, respectively; Figure 5a). Likewise, in HMC3 cells, the levels of NLRP3 increased by 10.4%, 25.7%, and 64.2% at 1 mM sucralose concentration for 7, 14, and 21 days, respectively, compared to the control group (*p* = .4885, *p* = .015 and *p* < .0001, respectively; Figure 5b). The study showed that treatment with 1 mM sucralose concentration for 7, 14, and 21 days in HMC3 cells led to significant increases in 8‐OHdG levels, a marker of oxidative DNA damage. Notably, the 8‐OHdG levels increased by 4.8% after 7 days, 19.4% after 14 days, and 51.5% after 21 days of sucralose treatment compared to the control group (*p* = .1758, *p* = .0004 and *p* < .0001, respectively; Figure 5c). Furthermore, the sucralose‐treated HMC3 cells also exhibited higher levels of MDA, a product of lipid peroxidation. The MDA levels increased by 8.3% after 7 days, 25.2% after 14 days, and 61.8% after 21 days of treatment with 1 mM sucralose compared to the control group (*p* = .679, *p* < .0001, and *p* < .0001, respectively; Figure 5d). These findings suggest that long‐term sucralose treatment negatively regulates neuroinflammation, DNA damage, and lipid peroxidation in HMC3 cells.

The ELISA analysis revealed that sucralose treatment in HMC3 cells led to significant reductions in GPx4 levels, a key marker of ferroptosis. Specifically, GPx4 levels decreased by 12.6% after 7 days, 27.5% after 14 days, and 58.3% after 21 days of treatment with 1 mM sucralose compared to the control group (*p* = .0132, *p* < .0001, and *p* < .0001, respectively; Figure 5e). This indicates that sucralose treatment over time negatively affects GPx4 expression, which could contribute to the promotion of ferroptosis in these cells. Moreover, the levels of GSH, an important antioxidant that plays a critical role in protecting cells from oxidative stress, were also significantly reduced in HMC3 cells treated with 1 mM sucralose for 7, 14, and 21 days. GSH levels decreased by 14.7% after 7 days, 35.8% after 14 days, and 66.2% after 21 days of sucralose treatment compared to the control group (*p* = .0071, *p* < .0001, and *p* < .0001, respectively; Figure 5f). This suggests that sucralose treatment affects the cellular antioxidant defense mechanism by depleting GSH levels, further contributing to increased susceptibility to oxidative damage. Additionally, HMC3 cells treated with 1 mM sucralose for 7, 14, and 21 days exhibited a decrease in SIRT1 levels by 9.2%, 20.6%, and 43.4%, respectively (*p* = .0026, *p* < .0001 and *p* < .0001 vs. control, respectively; Figure 5g). SIRT1 is known for its antioxidant properties and its involvement in various cellular processes, including stress resistance and inflammation. The decrease in SIRT1 levels upon sucralose treatment could further impact the cellular response to oxidative stress and inflammation, possibly exacerbating the negative effects observed in the study. To assess whether sucralose induces ferroptosis by measuring Fe^2+^ levels, HMC3 cells were exposed to 1 mM sucralose for 7, 14, and 21 days. Fe^2+^ levels decreased by 6.1% after 7 days, 22.8% after 14 days, and 40.5% after 21 days of sucralose treatment compared to the control group (*p* = .027, *p* = .0006, and *p* < .0001, respectively; Figure 5h). The results provide insights into the potential effects of sucralose on cellular damage and ferroptosis in microglial cells, highlighting the importance of considering the impact of artificial sweeteners on neuroinflammatory processes and cellular health.

### 3.6 Effect of sucralose treatment on the expression levels of IL‐1β, NLRP3, SIRT1, and GPx4 in HMC3 cells

**FIGURE 6:**
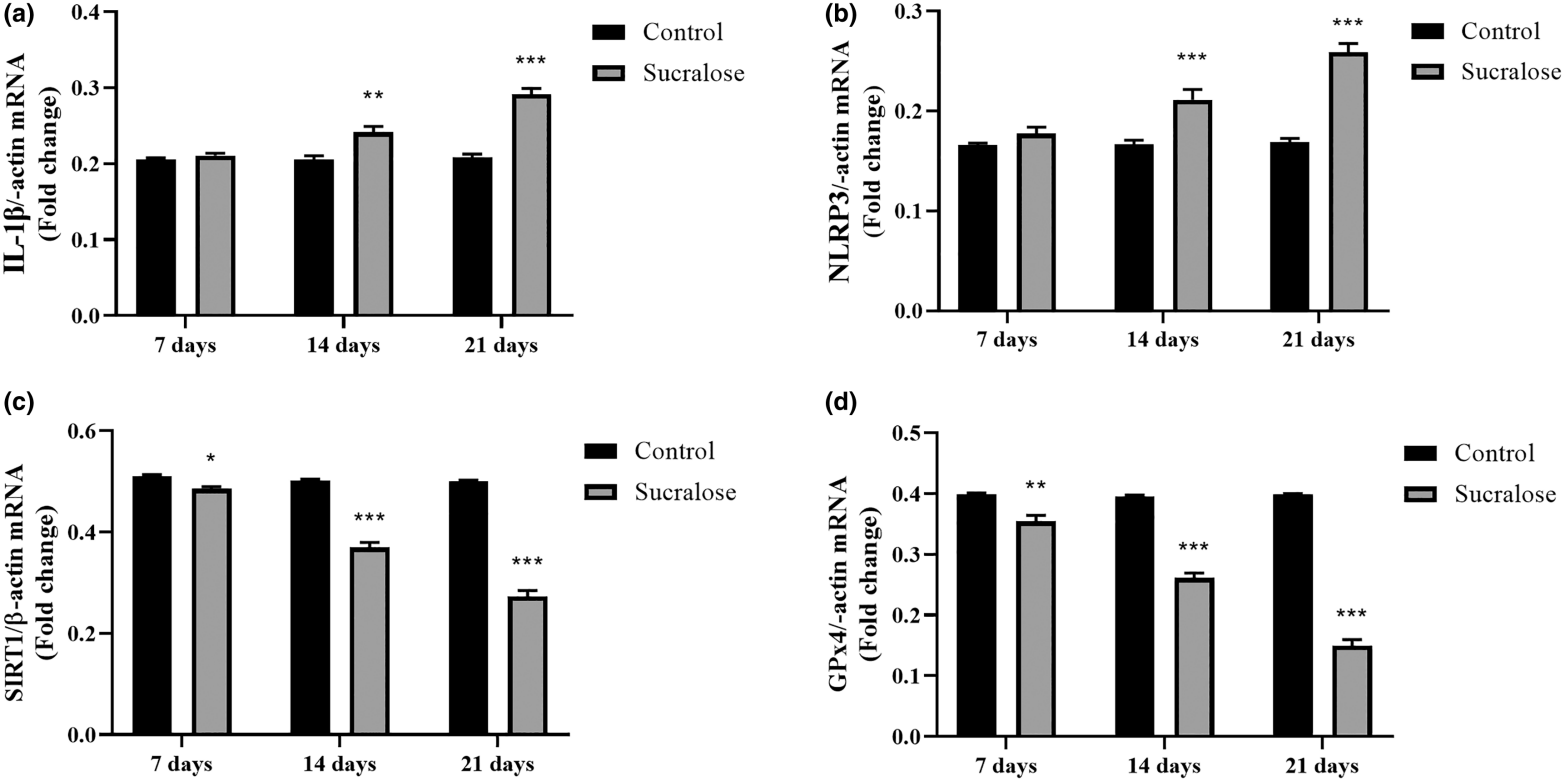
Effects of long‐term sucralose treatment on expression levels of IL‐1β, NLRP3, SIRT1, and GPx4 in HMC3 cells. (a) IL‐1β mRNA levels in HMC3 cells; (b) NLRP3 mRNA levels in HMC3 cells; (c) SIRT1 mRNA levels in HMC3 cells; (d) GPx4 mRNA levels in HMC3 cells. *p < .05, **p < .001, and ***p < .0001 vs. control group.

The quantitative real‐time polymerase chain reaction (qRT‐PCR) analysis presented in Figure 6 provides further insights into the gene expression changes induced by sucralose treatment in HMC3 cells. Consistent with the ELISA results, the mRNA levels of key inflammatory mediators IL‐1β and NLRP3 were significantly increased upon treatment with 1 mM sucralose for 7, 14, and 21 days. Specifically, IL‐1β mRNA levels in HMC3 cells increased by 8.2% after 7 days, 18.5% after 14 days, and 46.4% after 21 days of sucralose treatment compared to the control group (*p* = .165, *p* < .0001, and *p* < .0001 vs. control, respectively; Figure 6a). This suggests that long‐term exposure to sucralose induces a proinflammatory state in these cells, as evidenced by the upregulation of IL‐1β expression. Similarly, NLRP3 mRNA levels in HMC3 cells increased by 9.6% after 7 days, 31.5% after 14 days, and 59.1% after 21 days of sucralose treatment compared to the control group (*p* = .224, *p* < .0001, and *p* < .0001 vs. control, respectively; Figure 6b). The upregulation of NLRP3 indicates the activation of the NLRP3 inflammasome, a critical component of the inflammatory response, and further supports the induction of inflammation in HMC3 cells following sucralose exposure.

In contrast, the mRNA levels of SIRT1 and GPx4, two key components involved in antioxidant defense, stress resistance, and ferroptosis regulation, were significantly decreased in HMC3 cells following sucralose treatment. Specifically, SIRT1 mRNA levels decreased by 8.8% after 7 days, 32.5% after 14 days, and 58.4% after 21 days of sucralose treatment compared to the control group (*p* = .0167, *p* < .0001, and *p* < .0001, respectively; Figure 6c). This reduction in SIRT1 expression could lead to a decrease in cellular antioxidant capacity and stress response, potentially rendering the cells more vulnerable to oxidative damage and inflammation. Similarly, GPx4 mRNA levels decreased by 11.4% after 7 days, 33.7% after 14 days, and 61.5% after 21 days of sucralose treatment compared to the control group (*p* = .0011, *p* < .0001, and *p* < .0001, respectively; Figure 6d). Given GPx4's crucial role in protecting cells against lipid peroxidation and ferroptosis, its decreased expression suggests a compromised ability of HMC3 cells to counteract oxidative stress and lipid peroxidation in response to sucralose exposure. Collectively, the qRT‐PCR results provide strong evidence that sucralose treatment induces both an inflammatory response and enhances oxidant stress‐related ferroptosis mechanisms in HMC3 cells. These findings further support the notion that long‐term exposure to sucralose may have detrimental effects on microglial cells and their ability to maintain cellular homeostasis.

### 3.7 Effect of sucralose on the apoptosis profile in HMC3 cells

**FIGURE 7:**
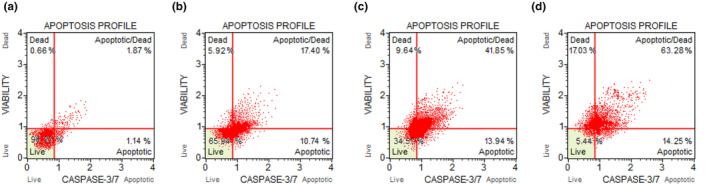
Long‐term sucralose treatment induced apoptosis in HMC3 cells. (a) Untreated HMC3 cells; (b) HMC3 cells treated with 1 mM sucralose for 7 days; (c) HMC3 cells treated with 1 mM sucralose for 14 days; (d) HMC3 cells treated with 1 mM sucralose for 21 days.

The results from the flow cytometry analysis presented in Figure 7 provide further evidence of the pro‐apoptotic effects of sucralose on HMC3 cells. Caspase‐3/7 activation, a key indicator of apoptosis, was analyzed to assess the impact of sucralose treatment on cell death pathways. In the control group, caspase‐3/7 activation was observed at a low level, with only 1.87% of HMC3 cells showing caspase‐3/7 activity (*p* > .05; Figure 7a). This low baseline level of caspase‐3/7 activation indicates that the control cells were predominantly in a non‐apoptotic state. However, treatment with 1 mM sucralose for 7, 14, and 21 days significantly increased caspase‐3/7 activation in HMC3 cells. After 7 days of sucralose treatment, there was a 17.4% increase in caspase‐3/7 activation compared to the control group (*p* < .0001; Figure 7b). After 14 days of sucralose treatment, the increase in caspase‐3/7 activation was more pronounced, reaching 41.85% compared to the control group (*p* < .0001; Figure 7c). Moreover, the most significant increase in caspase‐3/7 activation was observed after 21 days of sucralose treatment, with a 63.28% increase compared to the control group (*p* < .0001; Figure 7d). This indicates that the pro‐apoptotic effects of sucralose become more pronounced with prolonged exposure, and at this time point, a substantial proportion of HMC3 cells show signs of apoptotic activity.

## 4 DISCUSSION

Microglia, as resident macrophages in the CNS, play crucial roles in immune surveillance, synapse maintenance, and overall neuronal health (Sousa et al., 2017). They are highly responsive to changes within the brain and exist as heterogeneous populations, adapting to the specific homeostatic requirements of different CNS regions. Their responsiveness allows them to protect the brain from infection and effectively respond to extracellular changes (Gosselin et al., 2017). Microglia perform various vital functions by secreting cytokines, chemokines, growth factors, and neurotrophic factors (Greenhalgh et al., 2020). They also exhibit direct antimicrobial activity through processes like phagocytosis. The roles of microglia in inflammation regulation are often described using two primary phenotypes: M1 and M2 (Butler et al., 2021). In the M1 phenotype, microglia produce proinflammatory and neurotoxic cytokines, resulting in tissue damage, neurodegeneration, and cognitive impairment and contributing to ongoing inflammation. During the M1 process, the release of inflammatory factors such as TNF‐α, IL‐1β, IL‐6, reactive oxygen species (ROS), and nitric oxide (NO) contributes to the inflammatory response in the brain, which is a protective mechanism to remove harmful agents and restore homeostasis. However, excessive or prolonged neuroinflammation can have detrimental effects, leading to tissue damage, neurodegeneration, and cognitive impairment. Conversely, the M2 phenotype involves microglia secreting anti‐inflammatory mediators and neurotrophic factors, aiding in the restoration of brain homeostasis. Neuroinflammation plays a pivotal role in the pathogenesis of various neurological disorders, including neurodegenerative diseases, autoimmune conditions of the CNS, and brain infections (Ní Chasaide & Lynch, 2020). Therefore, regulating the release of inflammatory factors and neurotoxic substances represents a promising therapeutic strategy for managing neuroinflammation. In this study, our primary objective was to investigate the effects of sucralose on HMC3 cells and delve into the underlying mechanisms that lead to cell death. For the first time, our research demonstrated that sucralose induces cell death in HMC3 cells by activating neuroinflammatory responses and triggering ferroptosis. Moreover, the study sheds light on the underlying molecular mechanisms by identifying the involvement of specific signaling pathways, such as SIRT1, NLRP3, IL‐1β, GPx4, and Fe^2+^, which play critical roles in regulating inflammation, antioxidant mechanisms, and ferroptosis. Long‐term treatment with sucralose also significantly impacted cell proliferation in HMC3 cells. Through the modulation of the SIRT1/NLRP3/IL‐1β/GPx4 pathways, sucralose altered cell proliferation, further emphasizing its potential harmful effects on microglial cells. The inhibition of SIRT1 and GPx4, along with the induction of NLRP3 and IL‐1β, enhanced the cytotoxic effects of sucralose on HMC3 cells. These observations suggest that the identified signaling pathways may be critical mediators of sucralose‐induced cell death. These findings shed light on the potential interplay between sucralose‐induced cell death and the manipulation of specific molecular pathways, providing valuable insights for further understanding the impact of long‐term sucralose consumption on brain cells.

The relationship between artificial sweetener usage and an increased risk of cellular damage remains a contentious topic, with various studies yielding conflicting results. Numerous studies have demonstrated that dietary habits are crucial in supporting cognitive functions and mitigating neurodegenerative processes. Additionally, various phytochemicals and bioactive compounds have been shown to prevent the progression of neurodegenerative disorders (Bahbah et al., 2021; Grewal et al., 2021; Singh et al., 2023; Uddin et al., 2020). However, recent research suggests a potential association between the consumption of artificial sweeteners and a higher risk of developing tumors, particularly with higher levels of consumption over extended periods (van Eyk, 2015). In this specific study, researchers observed morphological changes in human cancer cells (Caco‐2 and HT‐29) exposed to high concentrations of artificial sweeteners, particularly concentrations exceeding 10 mM. The treated cells displayed flattened, granular, and enlarged appearances with multiple nuclei, in contrast to untreated controls. Furthermore, the findings from this study demonstrated that both sodium saccharin and sucralose caused DNA strand breaks, as evidenced by increased comet tail length and percentage of DNA within the tails. It is important to note that the observed alterations in cell morphology after treatment with concentrations exceeding 10 mM of artificial sweeteners may also be significantly influenced by changes in pH and high osmolarity (Scott et al., 1991). In contrast, Rocha et al.'s (2011) study reported no morphological changes in red blood cells after exposure to 10 mM sucralose. However, a key difference is the limited exposure time to sucralose in their experiment, which was only 1 h. This short exposure time may not have allowed sufficient time for significant alterations in red blood cell morphology to become apparent. Additionally, Brusick et al.'s (2010) study, using various genotoxicity tests, did not find evidence of sucralose causing DNA damage or inducing mutations. These results contrast with the earlier study. Our results demonstrate that sucralose treatment caused a concentration‐ and time‐dependent reduction in cell viability in HMC3 cells. Low concentrations of sucralose (1–10 mM) did not significantly affect cell viability during the initial 24 h, but higher concentrations (20 and 50 mM) led to a progressive decrease in cell viability over time (48 and 72 h). The most significant reduction in cell viability was observed after 72 h of treatment with a 50 mM sucralose concentration. Moreover, we also found that long‐term exposure (7, 14, and 21 days) to low concentrations (1 mM) of sucralose suppressed cell viability and proliferation and increased 8‐OHdG levels, causing DNA damage. These findings highlight the potential detrimental effects of sucralose on HMC3 cell viability, especially at higher concentrations and longer exposure durations.

The inactivation of the cellular antioxidant system, particularly the cystine‐glutamate antiporter (system Xc‐)/GPx4 antioxidant system, is a crucial mechanism in ferroptosis. GPx4, a key enzyme, plays a critical role in reducing lipid peroxides, organic hydroperoxides, and hydrogen peroxide utilizing GSH (Seibt et al., 2019). When there is a depletion of GSH or inactivation of GPx4, the cell cannot counteract lipid peroxidation, resulting in the accumulation of harmful ROS (Skouta et al., 2014). Consequently, the loss of GPx4 function or low expression of GPx4 becomes a crucial downstream step in the progression of ferroptosis. In a previous study, long‐term exposure to aspartame, an artificial sweetener, resulted in decreased levels of GSH, GPx, and glutathione reductase in the brain tissue of rats, along with increased MDA levels, thereby altering the oxidant/prooxidant balance (Ashok & Sheeladevi, 2014). Similarly, Adaramoye and Akanni (2016) demonstrated that administering increasing doses of aspartame for 9 weeks increased lipid peroxidation in the brain, kidney, and liver tissues of rats while decreasing the activity of antioxidant enzymes such as superoxide dismutase, catalase, and GPx. Additionally, as Fe^2+^ converts to ferric iron (Fe^3+^) through the Fenton reaction, it triggers heightened production of lipid peroxides and hydroxyl radicals, damaging cell membrane integrity and ultimately inducing ferroptosis (Dixon et al., 2012). In this study, we demonstrated that sucralose treatment significantly affects cellular redox, iron homeostasis, and lipid peroxidation in HMC3 cells. Specifically, sucralose treatment led to ferroptosis by decreasing the levels of Fe^2+^, GPx4, and GSH in the cells. Moreover, sucralose treatment resulted in increased levels of lipid peroxidation products and DNA damage, as evidenced by elevated levels of MDA and 8‐OHdG.

Inflammasomes are molecular complexes located within the cytosol that play a critical role in sensing various stimuli and triggering inflammatory responses as part of the innate immune defenses (Jiao & Gong, 2020). In the CNS, various cell types, including microglia, neurons, and astrocytes, express components of the inflammasome. Emerging evidence suggests that the activation of the NLRP3 inflammasome is implicated in neuroinflammation and neurodegenerative conditions (Albornoz et al., 2018). SIRT1 is a histone deacetylase that modulates signal transcription, cell apoptosis, oxidative stress, and inflammation by deacetylating intracellular signaling molecules. The overexpression or activation of SIRT1 has been shown to play a protective role in various nervous system injuries (Wei et al., 2021). In cellular experiments, resveratrol, an SIRT1 activator, has been demonstrated to suppress the release of inflammatory cytokines such as IL‐1β, IL‐6, and TNF‐α in BV2 microglial cells induced by amyloid‐beta (Aβ) (Feng & Zhang, 2019). This effect is achieved through the inhibition of the NLRP3 and NF‐κB signaling pathways. Moreover, numerous researchers have investigated the effects of SIRT1 activation in animal models of brain injury (He et al., 2017; Molaei et al., 2021; Zou et al., 2018). These studies have consistently shown that SIRT1 activation inhibits the release of inflammatory cytokines IL‐1β, IL‐18, and TNF‐α by suppressing the NLRP3 inflammasome signaling pathway. The NLRP3 inflammasome can trigger a cascade of events that lead to oxidative stress and DNA damage, ultimately resulting in p53 activation. P53 is a critical tumor suppressor protein that plays a central role in regulating cell cycle arrest, DNA repair, and programmed cell death (Licandro et al., 2013). Recent studies have reported that long‐term administration of aspartame increased lipid peroxidation in the livers of mice, leading to heightened NLRP3 activation, p53 induction, and DNA damage (Finamor et al., 2021). However, the underlying molecular mechanism of the SIRT1/NLRP3 axis in neuroinflammation remains incompletely understood. According to our data, long‐term sucralose treatment leads to an increase in IL‐1β levels in HMC3 cells. This elevation in IL‐1β is correlated with decreased SIRT1 activity and expression. Due to reduced SIRT1 activity and expression, NLRP3 levels increase, thereby promoting microglial activation. The findings from our study suggest a potential link between sucralose exposure, alterations in SIRT1/NLRP3 axis activity, and subsequent inflammatory responses in HMC3 cells. Furthermore, this study aligns with the previous findings mentioned earlier, indicating that sucralose‐treated cells experience increased DNA oxidation, leading to cell death. This observation was accompanied by an increase in NLRP3 levels and a decrease in SIRT1 levels.

## 5 CONCLUSION

Overall, the data suggest that high concentrations or long‐term sucralose exposure lead to a complex interplay of molecular events involving increased DNA oxidation, altered levels of NLRP3 and SIRT1, and subsequent microglial activation and cell death. Furthermore, sucralose treatment disrupts the cellular redox balance by reducing antioxidant defenses (GPx4 and GSH) and increasing lipid peroxidation products, resulting in elevated levels of ferroptosis and oxidative stress in HMC3 cells. These findings contribute to our understanding of the potential impact of sucralose on cellular processes and neuroinflammation, underscoring the importance of further research to fully elucidate the underlying mechanisms.

## AUTHOR CONTRIBUTIONS

**Ceyhan Hacioglu:** Conceptualization (equal); data curation (equal); formal analysis (equal); funding acquisition (equal); investigation (equal); methodology (equal); project administration (equal); resources (equal); software (equal); supervision (equal); validation (equal); visualization (equal); writing – original draft (equal); writing – review and editing (equal).

## FUNDING INFORMATION

None.

## CONFLICT OF INTEREST STATEMENT

The author declares no competing interests.

## Data Availability

Data will be made available on request.
